# Coronectomy of lower third molars in intimate relation to the inferior alveolar nerve: A report of two cases

**DOI:** 10.4317/jced.60335

**Published:** 2024-02-01

**Authors:** Ángel-Orión Salgado-Peralvo, Naresh Kewalramani, David Madruga-González, Alvaro Garcia-Sanchez, Javier Barbi-Actis, Mario Pérez-Sayáns

**Affiliations:** 1 ORCID: 0000-0002-6534-2816. DDS, MSc, MPH, PhD. Department of Dental Clinical Specialties, Faculty of Dentistry, Complutense University of Madrid, Spain; 2 ORCID: 0000-0002-8174-0937. DDS, MSc. Department of Nursery and Stomatology, Faculty of Dentistry, Rey Juan Carlos University, Spain; 3 ORCID: 0000-0002-5162-2841. DDS, MSc. Department of Nursery and Stomatology, Faculty of Dentistry, Rey Juan Carlos University, Spain; 4 ORCID: 0000-0003-1235-9400. DDS, MSc. Department of Oral Health and Diagnostic Sciences, School of Dental Medicine, University of Connecticut Health, USA; 5 DDS, MSc. Department of Surgery and Medical-Surgical Specialties, Faculty of Dentistry, University of Santiago de Compostela, Spain; 6 ORCID: 0000-0003-2196-9868. DDS, MSc, PhD, PhD. Oral Medicine, Oral Surgery and Implantology Unit (MedOralRes), Faculty of Medicine and Dentistry, University of Santiago de Compostela 15782, Spain. Instituto de Investigación Sanitaria de Santiago (IDIS), ORALRES Group, Santiago de Compostela, Spain

## Abstract

Damage to the inferior alveolar nerve (IAN) secondary to the extraction of the lower third molar (LTM) is a relatively frequent complication (0.35–8.40%) that can cause temporary or permanent nerve damage. Coronectomy has been proposed as an alternative, which consists of sectioning the coronary portion of the LTM, and deliberately leaving the radicular portion with the pulp intact. Two clinical cases are presented in this article, in which root migration (0–0.3 mm) and a change of angulation (+2º to +9°) occurred. None of the cases developed complications during the follow-up period (12 months). Therefore, coronectomy is a procedure to be considered in selected cases as an alternative to conventional exodontia of the LTM to avoid possible damage to the IAN.

** Key words:**Case report, third molar, mandibular third molar, coronectomy, mandibular nerve, mandibular nerve injuries, root migration.

## Introduction

Damage to the inferior alveolar nerve (IAN) as a result of lower third molar (LTM) extraction is a relatively frequent complication (0.35-8.40%). The nature of the alteration is variable and includes hypoesthesia, paresthesia, hyperesthesia or even dysesthesia of the lower lip, the skin over the chin, the teeth and gingivae on the affected side. These alterations are temporary in 96% of cases, resolving after 4-8 months. However, in 2.10% of cases, they become permanent (1). Other complications resulting from damage to the IAN include functional problems, a reduction in the patient’s quality of life and medico-legal claims. In this regard, “coronectomy” or “deliberate root retention” is a surgical technique aiming to section the coronary portion of the LTM, thus leaving the root portion intact to reduce possible damage to the IAN. Therefore, LTM coronectomy could be considered when LTM removal is indicated, such as in cases of associated pathologies, such as carious and/or periapical lesions, recurrent pericoronitis, cystic/neoplastic lesions, and second molar lesions where the LTM is closely related to the IAN and complete tooth removal may result in nerve damage. Some radiological signs predictive of IAN damage are canal deviation at the apex, the presence of the juxta-apical area (i.e., a radiolucent area well-circumscribed laterally to the root and not at the apex) (2), interruption of the white lines of the canal, and narrowing or deviation of the root (3).

Despite this, controversy persists regarding this procedure since leaving the roots included is perceived as a risk of late complications (4), as it has been hypothesized that this might induce pulpitis and necrosis of the pulp tissue and, consequently, osteomyelitis, a root cyst, or apical periodontitis that could lead to an infection affecting the IAN (5). In cases of recurrent pericoronitis where the LTM has partially erupted and has sufficient space to enter occlusion, an alternative may be “operculectomy”, which is a minor surgical procedure in which a small flap of tissue is removed over the partially erupted tooth, creating an environment that prevents plaque accumulation and subsequent inflammation (6).

The present article aims to present two clinical cases in which coronectomy of LTMs was performed given the associated high risk of damage to the IAN and mandibular fracture.

## Case Report

-Diagnosis

The initial clinical presentation in all cases was recurrent pericoronitis. Initial diagnostic orthopantomography (OPG) and cone beam computed tomography (CBCT) (Planmeca Romexis®), were both performed to confirm the intimate relationship of the LTM apexes with the IAN canal. After informing each patient and, in the case of minors, their legal guardian, about the associated risks of nerve damage and mandibular fracture following conventional full exodontia of the LTM, they provided informed consent for the coronectomy procedure.

-Surgical technique

All procedures were carried out under local anaesthesia, using local infiltrative anaesthesia with articaine hydrochloride/epinephrine (Ultracain® 40/0.01 mg/mL, Normon™). A conventional mucoperiosteal buccal flap with a releasing incision was elevated. In this regard, it is recommended not to separate the lingual flap, but rather to protect it from surgical manoeuvres using a periosteal elevator. A buccal and distal osteotomy was performed with piezoelectric instruments (Surgic Touch LED®, Woodpecker™ with a UL3 tip) to access the cementoenamel junction and section the coronary portion of the LTM horizontally with a handpiece with a 701 tapered fissure bur. Any sharp fragments of retained tooth structure are smoothed down with a 2.3 mm diameter round diamond bur with copious saline irrigation, at least 3 mm below the ridge of the lingual and buccal plates. A critical factor is not to luxate the LTM at any time. No attempts were made at root canal treatment or any other therapy for the exposed vital pulp of the LTMs.

**Figure 1:**
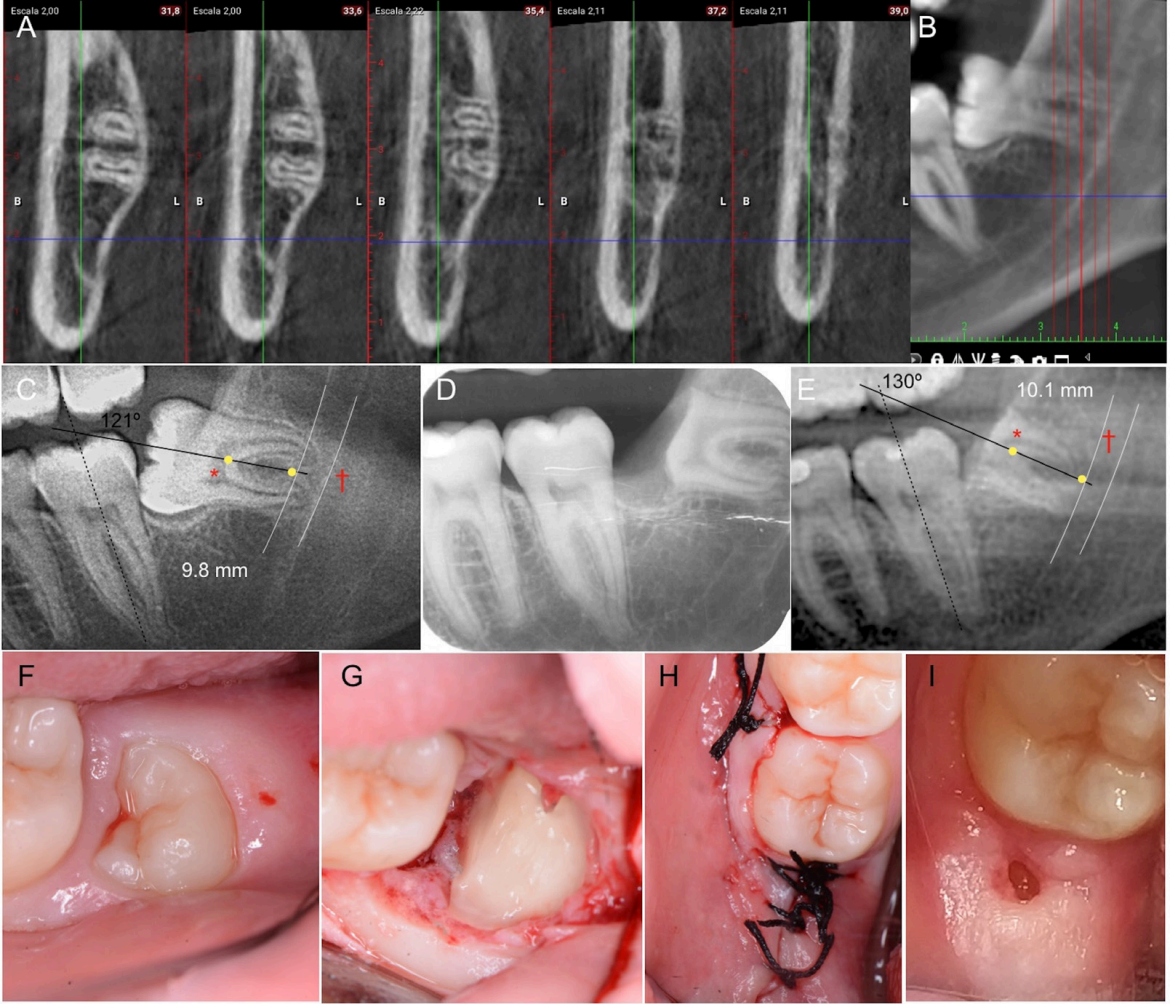
Case no. 1. Initial CBCT. A) Coronal sections separated at 1.80 mm; B) Sagittal section. C) Initial OPG, (D) immediate postoperative periapical, (E) OPG at 12 months follow-up (*Stable reference [most apical part of the pulp chamber] and †point where the axial axis of the LTM contacts the upper part of the IAN canal). F) Initial intraoral clinical image of 3.8.; G) Coronectomy of 3.8. H) Immediate postoperative phase; I) Operculitis diagnosed at 12 months follow-up due to coronal migration of the root portion.

**Figure 2:**
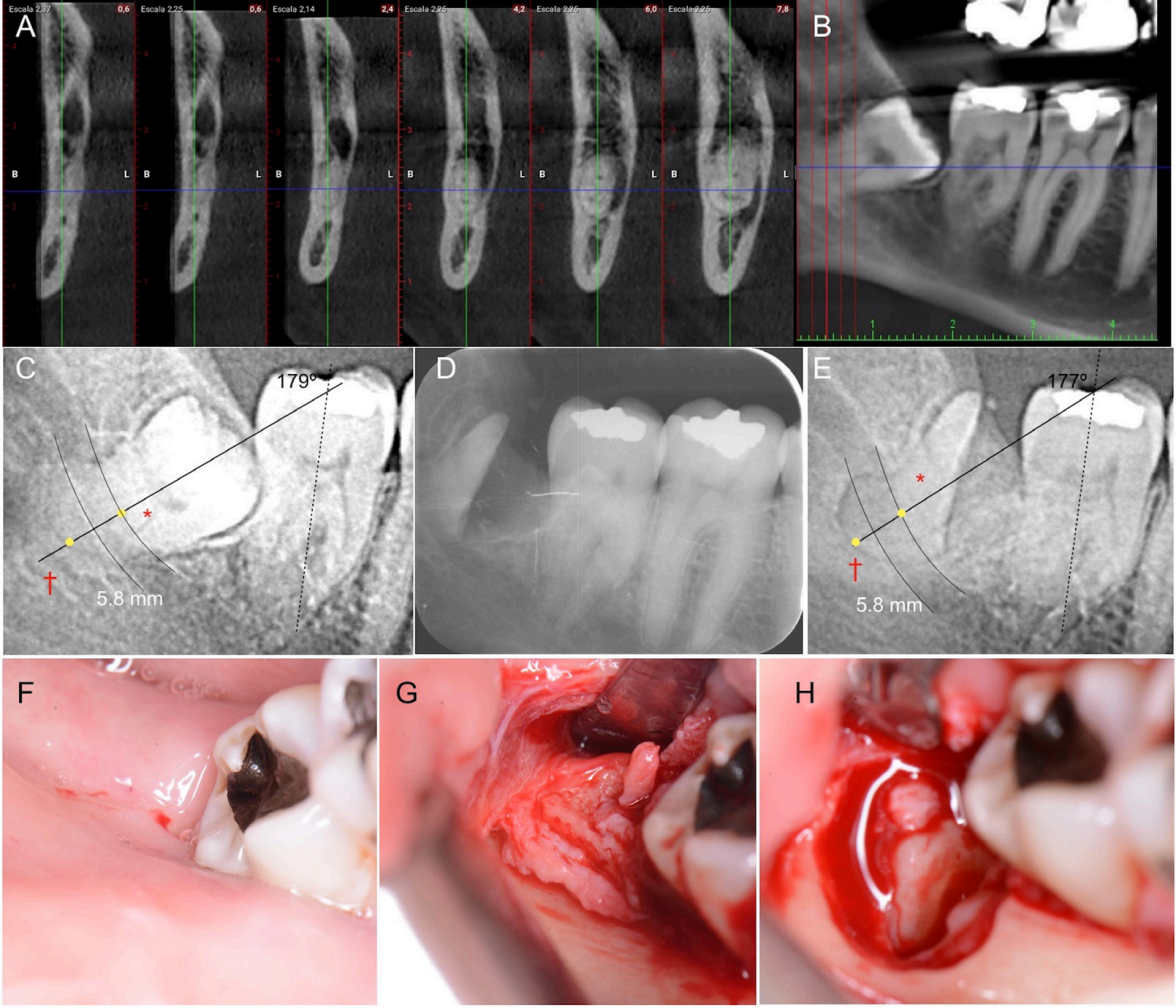
Case no. 2. Initial CBCT. A) Coronal sections separated at 1.80 mm; B) Sagittal section. C) Initial OPG, (D) periapical in the immediate postoperative period, (E) OPG at 13 months follow up (*Stable reference [upper part of the IAN canal] and, †most apical point of the radicular portion in the axial axis of the LTM). F) Initial intraoral clinical image of 4.8. covered by mucosa; G) Buccal mucoperiosteal flap elevation and protection of the lingual flap; H) Coronectomy of 4.8.

Following the procedure, a control periapical radiograph was taken (Soredex Digora™ Optime) and patients were advised to rinse with chlorhexidine digluconate 0.12% + cetylpyridinium chloride 0.05% (PerioAid® treatment, Dentaid™) every 12 h for 15 days. Radiographic images of the LTM from the initial OPG and the maximum follow-up period were superimposed using the radiographic subtraction technique to measure, using sTable reference points, the possible migration of the remaining root portion, as well as changes in its angulation. No infectious complications were observed during the follow-up period (12 months). Specific details of each case can be found in Table 1 and Figs. 1 and 2.

**Table 1:**
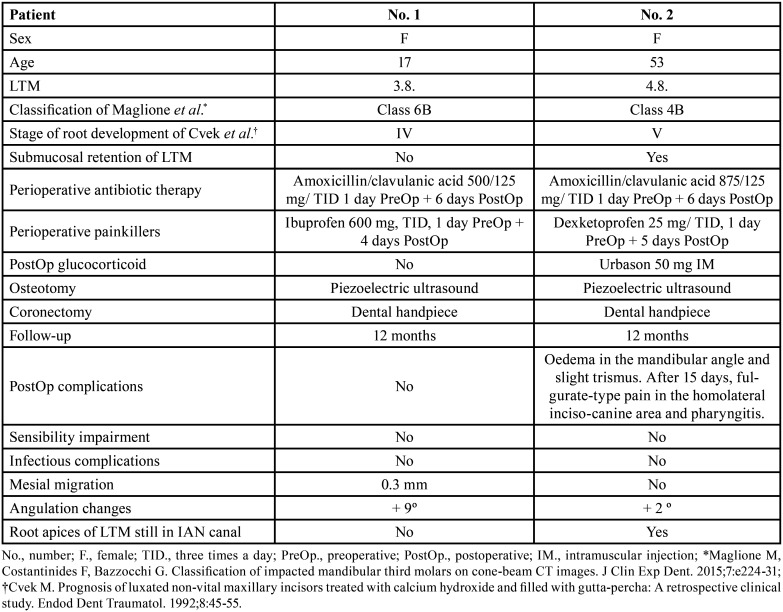
Characteristics of the included patients.

## Discussion

In the described cases, the pulp of the LTMs remained intact. Animal model studies have shown that pulp vitality is preserved with minimal degenerative changes, and the osteocementum typically extends to cover the exposed root portion (7), human studies have shown a higher rate of complications in coronectomies where root canal treatment was performed. When the pulp is left intact, the early postoperative infection rate ranges from 0 to 5.8% (4).

Another complication described is root migration of the residual fragment, with a wide incidence (8.6-90.9%) given the highly variable follow-up periods (6 months to 10 years) (4). The most significant migration occurs within the first year, with a subsequent stabilization after 24 months (8). Some authors have attributed the migration of the decoronated tooth to the preservation of pulp vitality (9). On the other hand, root migration is more strongly related to the female sex compared to the male sex (6.00 ± 3.34 mm vs. 3.78 ± 2.14 mm, respectively; *p*=0.002) (1) and a decline in migration is noted with increasing age (approximately 0.203 mm less per year of age increase) (8). Coronectomies are typically avoided in LTMs with immature apexes because of the increased risk of mobility during the procedure and the risk of eruption after the procedure (10). Other factors such as the status of eruption (*p*=0.232), the pattern (*p*=0.058) and depth of impaction (*p*=0.408), and/or root form (*p*=0.432) were not related to root migration (8).

Root eruption in the oral cavity is very rare (0–2.3%) – even in those cases where it reaches the crest – as the absence of occlusal forces in the retromolar zone allows the soft tissue on the alveolar crest to resist root eruption (11). Despite this, in case no. 1 operculitis occurred at 12 months follow-up due to migration of the radicular portion (Fig. 1I). It was controlled by improved and careful hygienic measures and removal of the root fragment has not been necessary.

Some authors have suggested that the presence of enamel remnants after coronectomy may inhibit bone growth over the sectioned roots (12). This may be due to the fact that if the distance between the root and alveolar crest is ≥ 5 mm, the likelihood of the root portion being completely covered by bone rather than soft tissue is significantly higher (*p*<0.015) (11). Therefore, a higher probability of reintervention (9.52%) has been described to polish the enamel edges and avoid possible secondary patient discomfort, but not a higher risk of infection (4). The residual enamel in case no. 2 was not intentional (Fig. 2E), but a consequence of a reduced mouth opening and poor cooperation of the patient, which made the procedure difficult. In the early healing period (21 days after surgery), there was fulgurating pain in the homolateral incisor-canine area, but after the prescription of a multivitamin complex, this ceased. Whenever possible, it is recommended to completely section the crown.

A systematic review estimates the reintervention rate at 0–8.33%, though in some instances, this was due to orthodontic considerations or patient requests, potentially leading to an overestimation of these Figures (4). In the event that reintervention is deemed necessary, a bone bridge or separation between the LTM apices and the IAN canal is consistently observed in all cases. This observation effectively eliminates the risk of nerve damage to the IAN (13). A systematic review and meta-analysis (14) demonstrated favorable outcomes of coronectomies of LTMs compared to conventional total extraction, in terms of lower probability of IAN damage (OR=0.16; *p*<0.001), namely 8 times less risk. Another systematic review supported these data by observing a rate of 0–0.98% versus 0–18.6%, respectively (*p*<0.05). These authors did not describe lingual nerve disturbance (4). Other advantages observed were a lower risk of dry alveolitis (OR=0.51; *p*=0.058), lower risk of post-surgical infections (OR=0.87; *p*=0.71) and less postoperative pain (OR=0.68; *p*<0.015). Furthermore, the cost-benefit analysis showed that coronectomies are 12% cheaper, considering that a CBCT would not be performed if the indication for coronectomy is established through an OPG. However, in the case of performing a CBCT, a coronectomy would still be 4% cheaper (14). Another advantage is that it significantly increases the amount of bone in the distal aspect of the second molar at follow-up periods >7 years (mean=3.2–3.5 mm; *p*<0.001) (15).

Despite its value in certain cases, there are some contraindications to consider. These include active infection of the LTM, especially when it affects the apical region; LTM with mobility, either before the intervention or if the tooth is luxated during the procedure, as it could act as a foreign object and be a source of infection or migration; and, finally LTM impacted horizontally along the IAN canal, as nerve damage could occur during coronal sectioning (13). In the authors’ view, coronectomy is perceived as a technically more complex procedure than conventional total extraction and it is recommended that experienced surgeons perform it, as improper execution could result in luxation of the LTM and possible secondary damage to the IAN.

Future lines of research should be directed towards the study of this technique with larger sample sizes and long-term follow-up in order to assess the possible rate of late complications.

## Conclusions

With the limitations inherent to the description of clinical cases, it can be concluded that coronectomy is a technique to be considered in LTMs where conventional exodontia would increase the risk of damage to the IAN. It is possible that in cases where the LTM has immature apices, the risk of migration of the decoronated root portion may increase. However, in the case of migration, its exodontia in the second stage would eliminate the risk of nerve damage as the root segment would consequently relocate away from the IAN canal.
